# A municipality implemented behavioural intervention to improve quality of life among older adults: protocol for a mixed-methods pilot case study

**DOI:** 10.1186/s40814-026-01795-w

**Published:** 2026-03-14

**Authors:** Kiran M. Gerhardsson, Christina Brogårdh, Åsa B. Tornberg, Ellen Hellblom, Steven M. Schmidt

**Affiliations:** Department of Health Sciences, Lund University, Lund, Sweden

**Keywords:** Complex intervention, Behavioural change, Environmental proactivity, Older adults, Usability, Quality of Life, Daytime outdoor walking

## Abstract

**Background:**

Ageing is associated with circadian rhythm sleep disorders, poor sleep at night, less physical activity and more time spent indoors, affecting the wellbeing of older adults. Their sleep and mood could benefit from daytime outdoor physical activity, exposure to daylight, better indoor lighting and sleep routines. However, changes to routines can be challenging depending on individual behavioural conditions (e.g. having the physical and cognitive capacity) and a supportive social and physical environment, such as a walk-friendly environment. To address these challenges, we have developed a complex behavioural intervention, delivered as a web-based course (‘Light, activity and sleep in my daily life’) that targets light-related behaviour, outdoor walking and sleep behaviour among community-dwelling older adults. The study aims to evaluate the usability, usefulness and acceptance of the intervention, the intervention outcomes and whether changes to routines are sustained. In addition, perceived enablers and inhibitors to daytime outdoor walking will be identified.

**Methods:**

We use a case study design and mixed methods because a deeper understanding of the real-world context is critical for a successfully implemented complex behavioural intervention. Eligible intervention participants (target *N* = 40) are Swedish-speaking adults (≥ 70 years), living in one-person households in apartments in four municipalities. Participants complete questionnaires assessing intervention outcome measures (e.g. quality of life), are interviewed about their daily routines, and wear an accelerometer which tracks activity and rest at the baseline. They then enrol in a 9-week course, including self-studies at home and four physical meetings at the senior citizen meeting point. Baseline measures are repeated directly after the course, at 3 and 10 months. In addition, participants evaluate the intervention’s usability and usefulness after the course and are interviewed at three months about perceived enablers and inhibitors to daytime outdoor walking. Staff from the municipality (a potential future service provider) assess their acceptance of the intervention delivery and discuss future implementation in focus groups (*N* = 4).

**Discussion:**

Results will inform a subsequent randomised study including a control group focused on optimising the intervention’s content and delivery procedures to enable an intervention better integrated into municipal health promotion services/strategies. An anticipated long-term outcome is continued independence.

**Trial registration:**

ClinicalTrials.gov Identifier: NCT06807060. Registered on 3 February 2025—retrospectively registered.

**Supplementary Information:**

The online version contains supplementary material available at 10.1186/s40814-026-01795-w.

## Introduction

### Background

Ageing is associated with circadian rhythm sleep disorders [1], poor sleep at night [2], less physical activity [3] and more time spent indoors [4]. In Sweden, more older adults (≥ 65 years) report having sleep problems (44%) than younger adults (39%), and it is more common among older women (53%) than older men (36%) [3]. A smaller proportion (54%) of older adults report being physically active 150 min/week compared to younger adults (64%) [3]. According to a study in Germany, people spend an average of 65% of their time indoors at home, but the figure for adults aged 65 and above is 81% [4].

Sleep and circadian rhythm disruption is associated with reduced cognitive abilities because sufficient sleep is critical for memory consolidation [1]. Other negative associations include, e.g. mood disorders, especially seasonal affective disorder [5]. One reason for circadian disruption could be reduced daytime light exposure among older adults. Physical inactivity can limit their exposure to daylight outdoors, and cataracts and age-related diseases can reduce the light signal to the body clock [1, 6]. Daytime light exposure has been shown to be too low to support sleep and wakefulness in healthy adults, according to recommendations [7]. Among older adults, daytime light exposure at home is too low for visual tasks and for stimulating a circadian rhythm [8, 9]. It is important to acknowledge that light exposure is highly determined by individual behaviour [10]. In shaping their indoor environment, many aspects will affect residents’ lighting choices, such as lighting preferences, cultural practices, chronotypes and health conditions. Awareness of light as the most important environmental time cue for the circadian clock, and knowledge of how to achieve appropriate lighting conditions in the home seem to be limited among Swedish residents [11]. There is a need for raising awareness and for improving daytime light levels, which could also be beneficial for reducing falls in the home [12, 13].

Ageing also involves changes in movement, both frequency and type of movement [14]. Previous research has identified many benefits of walking outdoors, e.g. improved health [15] and pleasure [16]. However, such benefits are dependent on both individual factors (motivation and capability) and environmental factors (e.g. opportunities supported by social and urban design, weather, latitude, air quality) [17–19]. A systematic literature review found a strong association between neighbourhood environmental characteristics and walking among older adults [20]. All studies included in the review were quantitative (published between 2000 and 2016), and a majority of the articles were published in North America, followed by Asia and Europe. A few studies were conducted in South America, Oceania and Africa. Only 4% were longitudinal (conducted in the USA).

Besides neighbourhood environment characteristics, other factors will impact older adults’ walking behaviour, including geographical location, topography and climate, in addition to local norms and regulations. During the past years, new vehicles have become more frequent on bicycle paths (and pavements), such as cargo bikes, electric bikes and electric scooters. E-scooters are banned or restricted in some cities because of pavement clutter or safety concerns.

Thus, there is a gap in the research literature regarding current perceptions of walkable neighbourhoods and the impact of other geographical locations on walking behaviours, beyond those included in the review. Furthermore, research on outdoor physical activity has not focused on perceived environmental factors enabling or hindering increased walking among older adults who are capable of walking outdoors or cycling in a Swedish context [21].

While many single-factor interventions address, e.g. either sleep or physical activity [22–24], no behaviour-change intervention has so far targeted the complex relationship between light, outdoor physical activity (in terms of walking) and sleep and how it impacts quality of life. Recently, associations between mobility and quality of life have been confirmed [25], as have the joint associations of physical activity and sleep duration with cognitive ageing [26]. Furthermore, little attention has been paid to research through design that examines the possibilities of self-modifications to improve the physical environment in ordinary homes of older adults who receive no or limited in-home care. To address these age-related challenges, we have developed a complex behavioural change intervention [27, 28]. The novelty lies in targeting light-related behaviour, outdoor walking and sleep behaviour, as well as self-modifications in the home. Guided by implementation science frameworks that highlight the importance of both the individual level and multiple ecological levels [29], we acknowledge the need to involve municipalities in this pilot study, given that they are potential providers of the intervention when it is implemented in the future.

### Development of the intervention: ‘Light, activity and sleep in my daily life’

The ‘Light, activity and sleep in my daily life’ (LAS) intervention directed at older adults was developed to promote wellbeing through increased physical activity, enhanced mood and sleep, and improved lighting and darkness conditions at home. The intervention focuses on health promotion through changes to routines (light-related behaviour, outdoor physical activity and sleep behaviour) and environmental proactivity. The latter refers to persons who modify their environments to live a healthy and independent life [30]. Environmental modifications include interior lighting, filtering daylight and blocking light at night, without risking accidents when getting up at night, and furniture arrangement.

The intervention is complex in that it considers multiple factors (e.g. light-related behaviour, physical activity and sleep behaviour) and components (e.g. cognitive goal setting and implementation [31, 32]), see Additional file 1. The intervention addresses self-identified needs, which can make it more effective [33].

The intervention is delivered as a web-based course on a digital platform and includes one introductory physical meeting and three additional physical meetings. Course material is placed in nine modules covering electric lighting, daylight, physical activity outdoors and sleep. Each completed module ends with a brief online evaluation. Besides online material, the course includes a test kit containing light bulbs, a sleep mask, a checklist for the room inventory, a cap, a notebook, and a sleep diary. The purpose of the test kit is to encourage experimentation and provide handouts and printed copies to facilitate the completion of assignments. Additional file 2 describes the intervention in more detail, based on the TIDieR checklist [34].

There are challenges related to the choice of strategy, i.e., a web-based course, to promote behavioural changes among community-living older adults, such as digital competence and Internet use in Sweden compared to other countries in the European Union. We reflect on those challenges in a previous article reporting on the development of the intervention [27].

In 2021–2022, usability evaluations of a first version were conducted in a full-scale model of an apartment by two sets of participants [27]: in a first round by experts and in a second round by pensioners representing the target users (community-dwelling adults aged 70 and over). Intervention content and design features were refined based on their feedback. In autumn 2022, intervention usability and study feasibility were evaluated in real-world homes by eight participants aged 71–84 [28]. The conclusion was that only minor changes to the intervention were needed based on participants’ feedback. Regarding the locality for the physical meetings, the researchers found the municipality’s senior citizen meeting point suitable for the purpose, and participants appreciated that meetings were at the same place. One finding was the need to extend the time for recruitment, and advertising in the local newspapers should be considered to reach a wider group of potential volunteers.

Based on the study findings from the field, we made the following design changes to the online intervention content: revised the weekly evaluation form so intervention participants can provide textual feedback to the course leader/interventionist; revised instructions for downloading the light meter app to the phone; and updated text links.

### Objectives

Here, we describe the protocol for a pilot case study aiming to evaluate the usability and acceptance of the LAS intervention, changes to routines and whether changes to routines are sustained. In addition, perceived enablers and inhibitors to daytime outdoor walking will be identified. Thus, our focus is to evaluate usability, usefulness, and acceptance, rather than to determine whether the intervention is effective in improving the intervention outcome measures.

The objectives are as follows (primary #1–4, secondary #5):

1. To evaluate the usability and usefulness of the intervention, that is, determine if the online intervention content is easy to use and the intervention is useful for older adults 70 and over.
2. To evaluate the acceptance of intervention delivery procedures (locality of physical meetings, recruitment) to municipal staff (potential service providers).
3. To identify motivation/capabilities/opportunities relating to outdoor physical activity (e.g. perceived enablers and inhibitors to daytime outdoor walking) and sleep routines.
4. To develop further training material for future course leaders/interventionists in dialogue with the municipal partners.
5. To gain insight into the potential effectiveness of the intervention in terms of activity and rest patterns, mood, sleep quality, behavioural skills and quality of life.

## Method

### Theoretical foundation

**Fig. 1:**
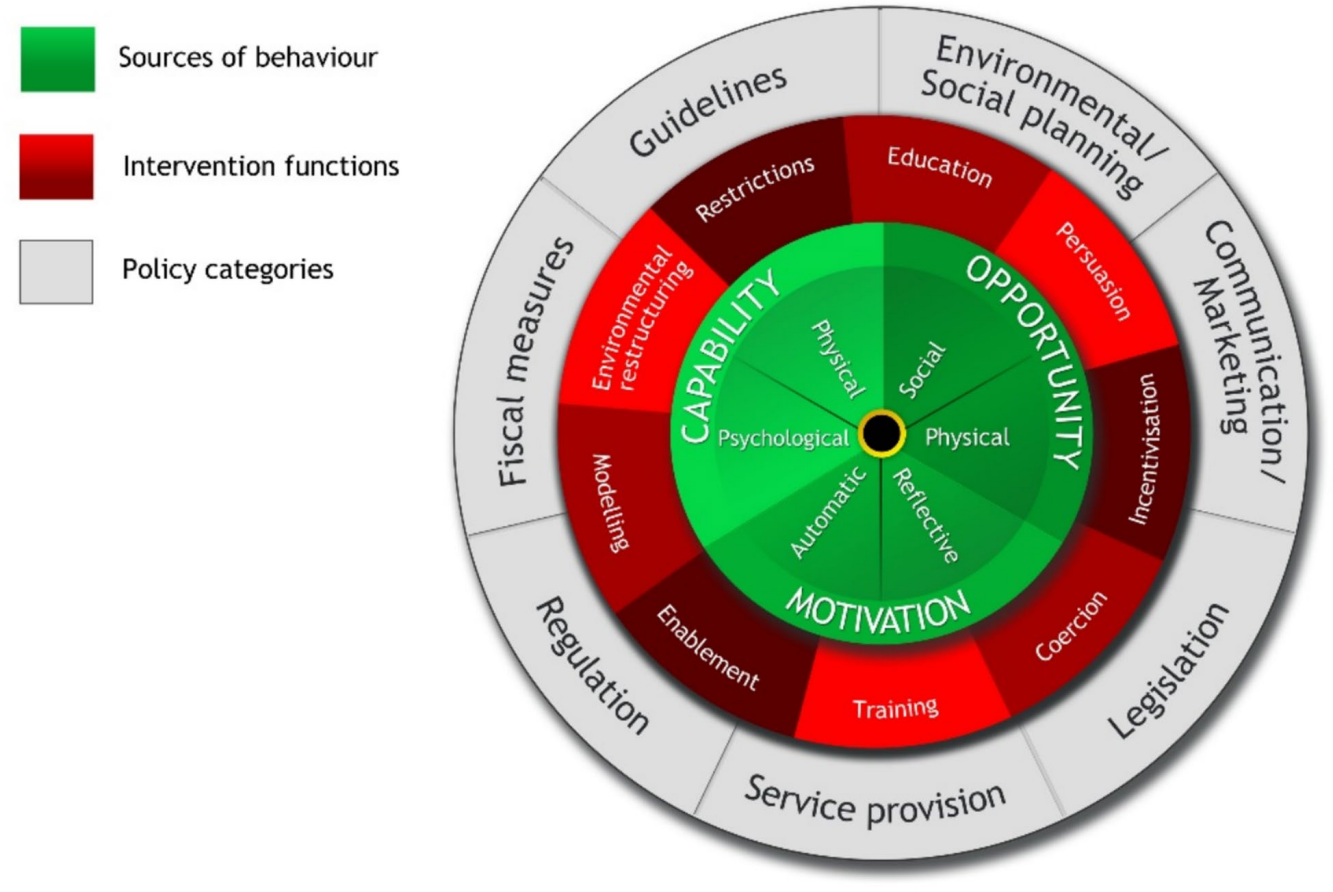
The Behaviour Change Wheel (reprinted with permission from Michie et al. [35])

On an overarching level, this interdisciplinary project builds on the framework of ‘The behaviour change wheel’ developed by Michie and colleagues [35] to conceptualise the critical elements when designing a health-related behaviour-change intervention (see Fig. 1). The framework is centred around a behaviour system consisting of three essential conditions: capability, opportunity, and motivation. Around the centre are nine intervention functions to handle deficits in capability, opportunity and/or motivation. Seven policy categories are placed along the outer circle, enabling the interventions to occur.

The project will include, but not be limited to, goal setting and implementation intention, emotion (Motivation) and knowledge (Capability). The project will also cover at least two intervention functions: changes to the physical environment and education. The project will address two policy categories: ‘Environmental/Social planning’ and ‘Service provision’.

**Fig. 2:**
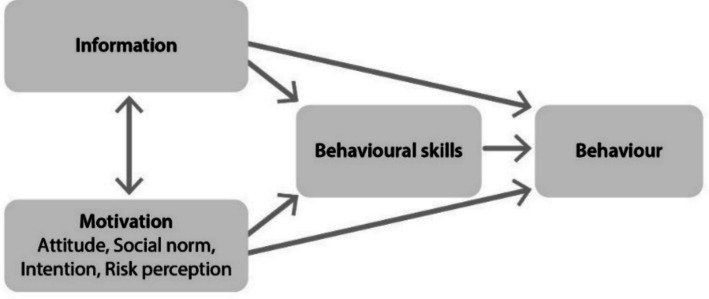
The original Information-Motivation-Behavioural Skills model (adapted with permission from Fisher & Fisher [37])

The intervention strategy departs from the Information-Motivation-Behavioural Skills Model, developed for promoting health-related behaviour while considering social and psychological factors that influence such behaviours [36]; see Fig. 2. According to the model, health-related information, motivation, and behavioural skills are fundamental for people’s health-related behaviours. When people are well informed, motivated to act, and have the behavioural skills needed for effective action, they will likely initiate and maintain health-promoting behaviours and experience positive health outcomes.

Factual information is provided about, e.g. light as the most potent external time cue for the internal body clock, characteristics of good indoor lighting, and the complex relationship between light, outdoor physical activity and sleep. Concerning motivation, the intervention includes information about the individual benefits of maintaining routines and weekly encouragement from the interventionist when each module is completed, and the intervention content is adapted to the target users. The intervention includes practical exercises and skills training to make learning experiences more interesting and enjoyable. Behavioural skills include, e.g. practising fundamental lighting design and sleep restriction, listing action plans to make goal-striving habitual behaviour. Behavioural changes involve physical activation (e.g. outdoor walking), changes to sleep routines and self-managed adjustments in the home.

Grounded in ‘The Behaviour Change Wheel’ framework and the aforementioned theoretical model, our proposition is as follows: if the intervention participants have the capability, are motivated, and have the opportunity, such as a supportive learning environment and the technical infrastructure, and a walk-friendly outdoor environment, then the LAS intervention could lead to the desired changes and be sustained over time. Capability refers to having the physical and cognitive capacity (acquiring information and practising learned skills). Motivation includes finding the intervention useful/relevant to one’s needs.

### A case study design

**Fig. 3:**
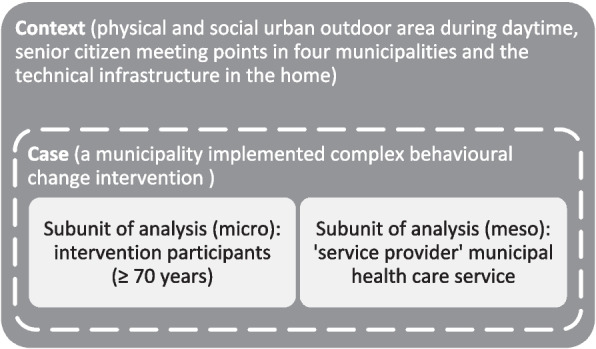
Type of design for the case study: a single case study with embedded subunits of analysis (adapted from the matrix in Yin [39])

This pilot study uses a single case study design with embedded subunits of analysis (see Fig. 3). The motivation for the design is to get a deeper understanding of the case (i.e., the principal unit of analysis) [38], which is a municipality implemented complex behavioural change intervention. Subunits of analysis are the individuals (older adults) who participate in the intervention and the municipal health care service as a potential service provider.

The context (i.e., data external to the case) is critical for successfully implementing the intervention in the future and involves: the physical and social urban outdoor area during daytime, senior citizen meeting points in the municipality and the technical infrastructure in the home. Because of the strong emphasis on the context, the research will be conducted in close collaboration with the municipalities. The final report will accordingly include a thorough description of both context and case study outcomes. Since it is a pilot study, the report will also explicitly address lessons learned in research design, field procedures and theoretical framing. We also intend to present recommendations for future implementation of the intervention—based on discussions with stakeholders—and for improvements of the urban outdoor environment to support daytime walking, backed up by study findings.

### Study sample and procedures

**Fig. 4:**
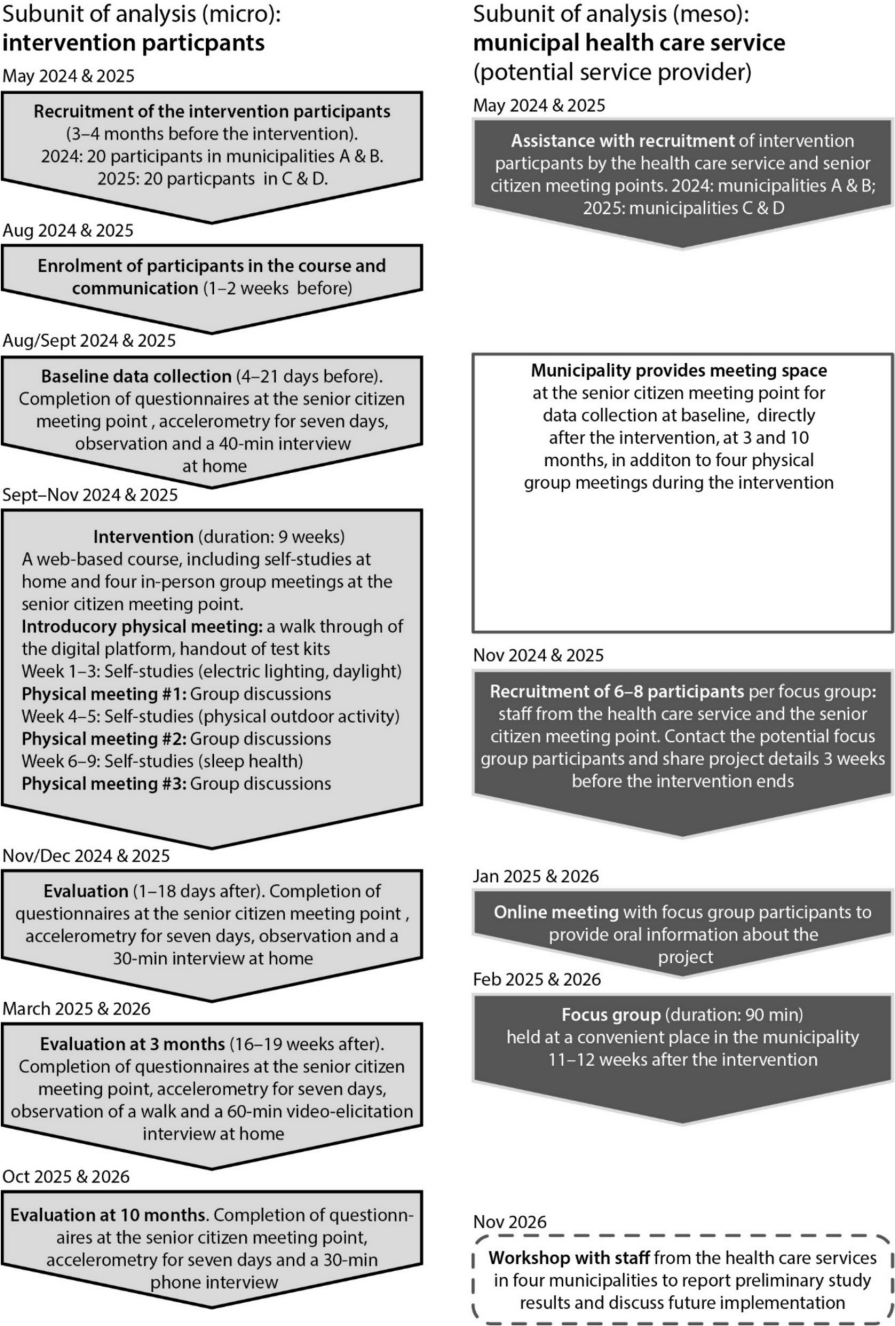
Study flow chart: data collection protocol, including subunits of analysis, materials and sources of material, and calendar months during which activities will occur. Data is collected in municipalities A and B, 2024–2025, and in municipalities C and D, 2025–2026

The intervention will be run as a single-arm open-label study. No control group is needed at this stage since the assessment of intervention outcome measures is secondary. Study procedures are outlined in Fig. 4. Four municipalities have been selected with similar characteristics, such as providing senior citizen meeting points, senior activities and minor home help services (named ‘Fixartjänst’ in Swedish). The participating municipalities (Lund, Malmö, Jönköping and Gothenburg) are inspired by or members of the WHO Global Network of Age-friendly Cities and Communities [40]. The geographical locations of the municipalities have also been considered to make the study viable.

Study participants eligible for the intervention are those aged 70 and over, ambulatory and sighted, Swedish speaking, living independently in one-person households in ordinary apartments and receiving no or limited home care services. The target group will likely have retired from work and experienced changes in daily routines. Living in apartments involves some challenges. Buildings and balconies can reduce access to daylight. Desired home modifications (e.g. lighting controls, interior or exterior solar screens) may be more challenging to make in rented apartments [41, 42].

After intervention delivery, staff from each municipality (6–8 per municipality) will participate in focus groups to discuss the acceptance of intervention delivery procedures and future implementation. Focus group participants will, for example, include staff from the senior citizen meeting point who have helped recruit intervention participants or assisted intervention participants at the senior citizen meeting points. Additional participants will include staff from the health care service and other divisions based on a dialogue with the health care service.

A detailed breakdown of recruitment, delivery of the intervention, data collection and communication of results is provided in Additional file 3.

#### Sample size justification

A convenience sample of 40 volunteers, 10 in each of the four municipalities, will participate in the intervention delivered as a web-based course, including physical meetings. Participants in two municipalities (A, B) took part in the intervention during autumn 2024, and the remaining participants in two additional municipalities (C, D) will take part during autumn 2025. Several factors were considered when selecting the sample size. First, the number of participants will be limited to ten in each municipality based on our experience from a previous usability study where a lower number was sufficient to identify usability issues [28]. Additionally, usability testing with five participants in qualitative usability studies has been shown to find a similar number of usability problems as one would find using many more test participants [43]. Another limiting factor is the available space for physical meetings at the senior citizen meeting point. A final consideration is the size of the group at the physical meetings. A group of up to ten participants is a suitable size for enabling discussions in which everyone can actively participate, given the duration of the meeting. Given the age group, we anticipate that some will discontinue the intervention due to life events or health reasons unrelated to the intervention. Still, a total sample size of 40 enrolled intervention participants will allow us to gain sufficient insight into the potential effectiveness of the intervention. It is recommended that at least 12 participants be considered for pilot studies [44], and the sample size should be large enough to provide useful information about the assessed aspects of the pilot study [45]. In a review of targeted and achieved sample sizes of pilot and feasibility studies, the median target sample size was 30 (IQR 20–50) [46].

Four focus groups are estimated to be adequate based on a study which concluded that more than 80% of all themes were discoverable within two to three focus groups and 90% within three to six focus groups [47]. These findings are applicable to the design of our focus groups because we also use a semi-structured interview guide, aim to have a homogenous sample (municipal staff working with older adults) and anticipate finding salient themes on a pre-determined topic (acceptance of intervention delivery procedures and future implementation).

### Data collection using a mixed-methods strategy for evaluations and assessment of success

A mixed-methods strategy of inquiry will be adopted [48]. The purpose of combining quantitative and qualitative data is to gain broader perspectives, which will allow for a deeper understanding of the acceptance of intervention delivery procedures (locality of physical meetings, recruitment) to the municipality, intervention participants’ perceptions of usability (e.g. perceptions of complexity, integration of functions, consistency), intervention usefulness and their motivation/capabilities/opportunities relating to outdoor physical activity (e.g. perceived enablers and inhibitors to outdoor walking). The joint analyses of quantitative and qualitative data will be used to decide whether the research should continue to the next step (randomised study with a control group), return to a previous step, repeat a step or stop the research [49]. We have included a few narrow criteria to determine whether the objectives have been achieved. However, the complexity of the intervention and how it interacts with the context in which it is implemented demands a broader analysis to inform our decisions to modify the intervention content or the study protocol.

A concurrent strategy will be used, that is, primary quantitative data and qualitative data will be collected at the same time by the researchers and later merged in the analyses of usability of the intervention, acceptance of intervention delivery procedures, and outdoor environmental characteristics enabling or hindering daytime outdoor walking. Collecting qualitative and quantitative data followed by analysis has several advantages: it reduces the burden on participants and facilitates their recollection. For example, the participants will complete a usability questionnaire after the intervention. During the same week, each participant will provide feedback in individual interviews, explaining in more detail any usability difficulties with the online intervention content. Combining questionnaire responses with verbal feedback will provide a more complete understanding. The final analysis will help determine whether our proposition holds: if the intervention participants have the capability (have the physical and cognitive capacity, can acquire information and practice learned skills), are motivated (that is, find the intervention useful/relevant to their needs), and have the opportunity (a learning environment and the technical infrastructure, a walk-friendly outdoor environment), then the LAS intervention could lead to the desired changes and be sustained over time.

The *usability* of the intervention, that is, if the online content is easy for the intervention participants to use, will be evaluated through a usability testing questionnaire (perceptions of complexity, integration of functions, consistency, see Additional file 4) and semi-structured interviews (see Additional file 5). Intervention usability will be considered acceptable if more than 50% of the intervention participants report a total usability score of 70 and above (out of 100), while considering participant feedback in the interviews.

Intervention *usefulness and relevance* to the participants will be evaluated through semi-structured interviews including questions about changes to routines and self-managed adjustments in the home. In addition, intervention *usefulness and relevance* to the participants will be indicated by measures of participant engagement (time logged on the course page, added participant comments in the weekly online evaluation after each module, intervention completion rate, observable self-managed changes in the home). Intervention usefulness will be considered acceptable if 70% of the intervention participants complete all nine modules of the web-based course, attend the first introductory course meeting to receive instructions for using the digital learning platform, attend at least one other physical meeting, and have made at least one change, either in the home or to routines relating to light, physical activity, or sleep. Participant feedback in the interviews will provide insights into the reasons for such changes and indicate whether the participants have applied what they learned.

The *acceptance* of intervention delivery procedures (locality of physical meetings, recruitment) to municipal staff (potential service providers) will be evaluated in focus groups. Discussion points will include identified challenges during delivery and thoughts on how the intervention could be integrated into municipal services in the future (see Additional file 6). Different options will be discussed to enable efficient delivery while considering contextual factors. The municipality’s acceptance of the intervention, which is partly carried out at a senior citizens’ meeting point, is anticipated to depend, for example, on whether the municipality’s senior citizens are interested in participating in the intervention and whether it adds an unreasonable staff workload. Feedback from staff in the interviews will provide insights into increased workload. In addition, we aim for at least 20 senior citizens attending the recruitment meeting, indicating sufficient interest.

*Motivation/capabilities/opportunities* relating to outdoor physical activity (e.g. perceived enablers and inhibitors to daytime outdoor walking) will be identified through video-elicited interviews with intervention participants to better understand circumstances enabling or hindering daytime outdoor walking (see Additional file 7). Video-elicitation interviews will use the following protocol: Intervention participants, who have completed the intervention, will be shadowed as they take one self-selected walk. The risk of falls and other potential walk-related hazards is assumed to be limited because participants will choose their walking routes and will not have to split their attention between walking and talking. Given the weather, the shadowed walks will be held in March to avoid the risk of icy conditions and snow. The researchers will record environmental features along the walk route (see Additional file 8), and the participants will video record the walk using body-worn cameras. Video recordings will be shown on a tablet in the participant’s home after the walk to assist recollection while the participant thinks aloud about the walk. Doing a face-to-face sit-down interview at home after the walk instead of during the walk has several benefits. This method enables more natural walks with no interruptions for conversations between the researchers and the participant during the walk. In addition, the method allows the researchers to record environmental features while walking. Playing the video will stimulate recall and elicit more information during the interview. The findings from the video-elicited interviews will inform designers and planners how opportunities for such activities can be improved to create more walk-friendly urban environments. The interview will end with additional questions about physical activity routines in general, sleep routines and time spent outdoors to identify any changes.

Plans to promote sustained engagement by the intervention participants and their completion of follow-ups include distribution of printed course and study activity schedules, scheduling home visits in good time before they take place, texting reminders of physical meetings, sending follow-up emails including text links to discussion topics raised by the participants at the physical meetings, and communicating study progress and expressing appreciation. To ensure the continued participation of the municipalities, we inform them of the study’s progress and issues raised by the intervention participants at the physical meetings if those issues are relevant to the municipalities.

To enable future implementation of the intervention, the training material for future course leaders/interventionists will be developed further based on an intervention delivery journal and field notes, and in dialogue with the municipal partners.

An overview of outcomes, methods of evaluation and when data is collected is provided in Tables 1 and 2.

**Table 1:** Primary outcomes: usability, usefulness and acceptance outcomes after intervention delivery, and possibilities for future implementation

Category	Method of evaluation	Reference	Timepoint
Usability	System Usability Scale questionnaire completed by intervention participants	[50]	1–18 days after
Technology acceptance (perceived ease of use and usefulness)	Semi-structured face-to-face interviews with intervention participants at home	[51]	1–18 days after
Acceptance of intervention delivery procedures and future implementation	Focus groups with intended service providers (municipal staff from the health care service and the senior citizen meeting point)	‘Checklist for high-quality implementation’ [52]	11–12 weeks after

**Table 2:** Primary outcomes (continued): Follow-up of changes to routines and opportunities after intervention delivery

Category	Method of evaluation	Timepoint
Changes to routines (indicating perceived usefulness)	Semi-structured face-to-face interviews with intervention participants at home	After, at 3 and 10 months
Self-managed changes in the home environment (indicating perceived usefulness)	Observations and semi-structured face-to-face interviews with intervention participants at home	After
Motivation/capability/opportunity for increased daytime outdoor walking, including perceived enablers and inhibitors to outdoor walking	Video-elicited part of the interview above	At 3 months
Environmental outdoor features along a walking route selected by the intervention participant	Observation by the researcher using a prepared form (Observer-based Environmental Assessment, OBEA)	At 3 months (before the video-elicited interview)

As this is a pilot study, it is not powered to detect any statistically significant changes in intervention outcome measures (activity and rest patterns, mood, sleep quality, behavioural skills, quality of life) (see Additional file 9). However, we will collect these data through questionnaires and accelerometry at baseline, after the intervention delivery, at 3 and 10 months to estimate potential changes in outcomes and whether changes are sustained and to determine whether the selected measures are appropriate.

### Data analyses

#### Quantitative data

Analyses of quantitative data measuring primary outcomes will be descriptive. Following published instructions on scoring of the 10-item System Usability Scale (SUS) [53, 54], a total usability score will be calculated ranging from 0 to 100 (SUS score). Higher SUS scores indicate greater usability. Based on empirical evaluations of the SUS, a SUS score below 50 indicates usability difficulties, while scores in the 70 s and 80 s are considered promising [55]. Missing values will be documented but not replaced with substitute values. The total time per week participants spend on the course site on the digital platform will be analysed descriptively. As previously stated, we anticipate that some will discontinue the intervention due to life events or health reasons unrelated to the intervention, but no more than 20%. Participants will be informed during recruitment that they may drop out without providing a reason.

Analyses of secondary quantitative data measuring repeated intervention outcomes (rest and activity patterns, mood, sleep quality, behavioural skills, quality of life) will be descriptive (see Additional file 9). Changes in outcome measures for the same group of intervention participants will be compared at a single time point before and after receiving the intervention, at 3 and 10 months. Missing data will be documented but not replaced with substitute values.

#### Qualitative data

Interviews and focus group discussions will be audio-recorded, summarised and analysed according to the following procedures.

Semi-structured interviews held at home at baseline including participants’ accounts of daily routines will be summarised into a graphical overview. Their accounts of readiness for change will be summarised in a table.

Semi-structured interviews held at home immediately following the intervention will be thematically analysed using a theory-driven approach based on the constructs of the Technology Acceptance Model [51]. Participants’ comments will be sorted into three pre-determined main themes: ‘Perceived ease of use’, ‘Perceived usefulness’ and ‘Course satisfaction’. Sub-themes and additional themes will be created inductively based on the participants’ comments.

The initial part of the semi-structured video-elicited interviews held at home at 3 months will be transcribed verbatim (participants’ description of their self-selected walk). Summaries and transcribed material will be analysed thematically using an inductive approach with first and second-cycle coding [56], after sorting participants’ comments into three main themes: ‘Motivation’, ‘Capability’ and ‘Opportunity’ (e.g. perceived enablers and inhibitors to daytime outdoor walking). The final semi-structured interviews on the phone at 10 months will also be analysed using a similar inductive approach. Only participants who complete the intervention will be included in the follow-up interviews.

Focus group discussions will be analysed thematically using an inductive approach with first and second-cycle coding [56].

## Discussion

Older adults may lack knowledge about their changing age-related needs and how light and physical activity during the day can affect sleep. The LAS intervention targets light-related behaviour, daytime outdoor walking and sleep behaviour directed at community-dwelling older adults living independently in one-person households. This pilot study is essential because it can provide knowledge to further the intervention and improve participant engagement. At the end of the project, intervention components will have been evaluated for usability and potential intervention effectiveness (i.e. improved quality of life). Intervention delivery will have been evaluated for acceptance. Also, opportunities for increased daytime outdoor walking will have been identified. In line with the case study approach, contextual conditions will have been analysed in relation to the case [39, 57, 58].

Given that the outcomes are as expected, the pilot case study will enable a more extensive evaluation of effectiveness in intervention outcome measures in the older adults taking part in the intervention in a future randomised study with a control group, including trained facilitators and a larger number of collaborating municipalities. Field notes and entries by the researcher in the intervention journal will provide an estimation of time required for future course leaders/interventionists and material for a training manual.

A strength of the pilot study is the close collaboration with potential service providers (municipal care service) who may deliver the intervention as a supplement to other municipal services to older citizens in the future. The study findings will show whether the intervention delivery procedures are acceptable to the municipalities.

One strength of the study design is the combined approach of architecture, psychology, physiotherapy and public health. Another strength is the mixed-methods approach using both quantitative and qualitative data to check the reliability of participant self-reports and interpret questionnaire results. For example, when participants show negative changes in mood or quality of life it might be because of poor sleep or life circumstances. Observations of participants’ self-managed home adjustments during the home visits after the intervention can confirm participants’ accounts. The combined use of self-reported questionnaire data and performance-based data (accelerometer-measured activity and rest patterns) further strengthens the study design. Self-reported time in the weekly online course evaluations can be compared with logged-in time on the digital learning platform.

One limitation is that the intervention participants in this pilot study may not fully represent the target sample, depending on their motivation for participating. It is anticipated that some volunteers will participate because they want to contribute to scientific research rather than because of an interest in behavioural change.

The absence of detailed progression criteria, such as a traffic-light system to specify them [59], can be considered a limitation of the study. However, as argued in the Methods section, the complexity of the intervention and its interactions with the context in which it is implemented demand a broader analysis to guide our decision on whether to modify the intervention content or the study protocol, or to move forward with a randomised study including a control group.

A limitation of using a case study design is that the results will be transferable only to municipalities with similar characteristics. However, including four municipalities will increase our understanding of what does and does not work when delivering this type of complex intervention. In this study, the intervention outcomes are secondary, and they will be evaluated in a future randomised study including a control group to enable conclusions about the intervention’s effectiveness. Detailed field procedures of the case study protocol and a training manual for the interventionist will enable repeated studies in the future.

## Conclusion

The findings will provide urban planners, consultants or municipal employees, and politicians in Sweden, as well as other countries with similar geographical and climate conditions, useful knowledge relating to the design and planning of urban outdoor areas. A more mindful environmental design can counteract various forms of inequality and physical barriers, for example, for older adults who want to go outdoors more during the day. Potential long-term outcomes are perceived safety through increased outdoor activity and social belonging, and inclusion through digital technology because the intervention participants will have gained more experience using the computer and the internet. Additional potential long-term benefits are increased self-efficacy and further improved quality of life.

## Supplementary Information

Additional file 1. Behaviour change techniques (BCTs).

Additional file 2. Intervention description based on TIDieR Checklist.

Additional file 3. Intervention breakdown and evaluation timeline.

Additional file 4. Usability testing questionnaire (SUS).

Additional file 5. Semi-structured interview guide (no. 1–2).

Additional file 6. Focus group interview guide.

Additional file 7. Semi-structured interview guide (no. 3–4).

Additional file 8. Environmental outdoor features form.

Additional file 9. Intervention outcome measures.

Additional file 10. SPIRIT Checklist.

## Data Availability

No datasets have been analysed. Data generated from the pilot study will be made available upon request.

## Abbreviations

BCT: Behaviour Change Techniques; OBEA: Observer-based environmental assessments
SUS: System Usability Scale
