# Supplementary material for: A municipality implemented behavioural intervention to improve quality of life among older adults: protocol for a mixed-methods pilot case study
Source: Pilot Feasibility Stud. 2026 Mar 14;12:47. doi: 10.1186/s40814-026-01795-w (PMC13063510; doi:10.1186/s40814-026-01795-w)
Supplement: Supplementary file 1 — Additional file 1. Behaviour change techniques (BCTs). [file 40814_2026_1795_MOESM1_ESM.pdf]

Additional file 1: Behaviour change techniques (BCTs)

The 'Light, activity and sleep in my daily life' intervention content includes several components labelled according to the Behavior Change Technique Taxonomy (Michie et al., 2013), as shown in Table 1.

Table 1. Intervention content ('Light, activity and sleep in my daily life').

**Category**[1]	**Component**	**Examples from the intervention**
Goals and planning	Goal setting (behaviour)	Self-selected goals, e.g., daily outdoor walks
	Problem solving	Participants are advised to identify barriers preventing them from changing routines, e.g., taking daily walks, and thinking of possible solutions, e.g., to walk with a friend
	Action planning	Participants are advised to plan beforehand when and where to act (implementation intention), e.g., taking an early morning walk directly after breakfast
Feedback and monitoring	Self-monitoring of behaviour	Self-recordings in sleep diaries and activity diaries
Social support	Social support	Face-to-face discussions with other participants at physical meetings to share experiences
Shaping knowledge	Instruction on how to perform a behaviour	On how to change sleep routines and increase the duration of daily walks
	Information about antecedents	On environmental and behavioural factors affecting sleep quality
Natural consequences	Information about health consequences	The effects of poor sleep quality on mood and individual functioning, environmental cues for setting the internal circadian clock
Regulation	Reduce negative emotions	Advice on accepting days of poor sleep or interrupted daily walk routines
Antecedents	Restructuring of the physical environment	Advice on basic lighting design to enable personalised changes to lighting and darkness conditions at home

[1] Categories including behavioural change techniques with similar active ingredients, i.e., by the mechanism of change (not the mode of delivery) (Michie et a., 2013).

Reference

Michie, S., Richardson, M., Johnston, M., Abraham, C., Francis, J., Hardeman, W., Eccles, M. P., Cane, J., & Wood, C. E. (2013). The behavior change technique taxonomy (v1) of 93 hierarchically clustered techniques: building an international consensus for the reporting of behavior change interventions. *Annals of behavioral medicine: a publication of the Society of Behavioral Medicine, 46(*1), 81–95. https://doi.org/10.1007/s12160-013-9486-6
