# Supplementary material for: A municipality implemented behavioural intervention to improve quality of life among older adults: protocol for a mixed-methods pilot case study
Source: Pilot Feasibility Stud. 2026 Mar 14;12:47. doi: 10.1186/s40814-026-01795-w (PMC13063510; doi:10.1186/s40814-026-01795-w)
Supplement: Supplementary file 2 — Additional file 2. Intervention description based on TIDieR Checklist. [file 40814_2026_1795_MOESM2_ESM.pdf]

Additional file 2: Intervention description based on TIDieR checklist

Article: A municipality implemented behavioural intervention to improve quality of life among older adults: protocol for a mixed-methods pilot case study

Authors: Kiran M. Gerhardsson[1], Ellen Hellblom[1], Christina Brogårdh[1], Åsa B. Tornberg[1], Steven M. Schmidt[1]

Department of Health Sciences, Lund University, Lund, Sweden[1]

1. Brief name	'Light, activity and sleep in my daily life' intervention to support changes to routines and self-managed changes in the home
2. Why? (rationale)	Indoor lighting, exposure to outdoor daylight, physical activity and sleep interact to influence functioning, mood and circadian rhythm. Older adults (≥ 65 years), who often spend more time at home, are less physically active and experience more sleep problems, could benefit from strategies to support changes to routines and self-managed modifications in the home. However, changing routines, such as increasing one's physical activity, can be challenging depending on individual behavioural conditions, e.g., having the physical and cognitive capacity (capability), finding the activity enjoyable and relevant to one's needs (motivation), and having a supportive social and physical environment (opportunity), such as a walk-friendly environment. Other challenges involve maintaining health-promoting physical activity and sleep routines over time.<br><br>To address these challenges, we have developed a complex behavioural intervention, delivered as a web-based course ('Light, activity and sleep in my daily life', LAS) that targets light-related behaviour, outdoor walking and sleep behaviour among community-dwelling older adults. The intervention focuses on multiple behaviours, such as physical activity outdoors and sleep routines, as well as environmental proactivity, referring to persons who modify their environments to live a healthy and independent life.<br><br>*Theory base*. The intervention strategy departs from the Information-Motivation-Behavioural Skills Model. Factual information is provided about, e.g., light as the most potent external time cue for the internal body clock, characteristics of good indoor lighting, and the complex relationship between light, outdoor physical activity and sleep. Concerning motivation, the intervention includes information about the individual benefits of maintaining routines and weekly encouragement from the interventionist when each module is completed, and the intervention content is adapted to the target users. The intervention includes practical exercises and skills training to make learning experiences more interesting and enjoyable. Behavioural skills include, e.g., practising fundamental lighting design and sleep restriction, listing action plans to make goal-striving habitual behaviour. Behavioural changes involve physical activation (e.g., outdoor walking), changes to sleep routines and self-managed adjustments in the home.<br><br>*Goal.* The intervention aims to promote wellbeing (e.g., better mood and sleep) and improve lighting and darkness conditions at home, so older people can continue living healthy and independent lives.
3. What? (materials)	The intervention is delivered as a web-based course on a digital learning platform. Course material is placed in nine modules covering electric lighting, daylight, physical activity outdoors and sleep. Besides online material, the

	course includes a test kit containing light bulbs, a sleep mask, a checklist for the room inventory, a cap, a notebook, and a sleep diary. The purpose of the test kit is to encourage experimentation and provide handouts and printed copies to facilitate the completion of assignments.
4. What? (procedures)	Eligible for the intervention are those aged 70 and over, ambulatory and sighted, Swedish speaking, living independently in ordinary apartments and receiving no or limited home care services. (The target group will likely have retired from work and experienced changes in daily routines.)<br>*Recruitment.* Municipal staff working at the senior citizen meeting points will be informed about the study so they can assist with recruiting participants for the intervention. Intervention participants will also be recruited through advertisements in the local newspapers and pensioner associations. Those who meet the inclusion criteria will be selected and receive a phone call from the research assistant to confirm background information (e.g., Swedish-speaking skills, if they have a laptop or a tablet, social security number to enable enrolment on the digital course). Invitations to meetings will be sent out and meetings will be organised.<br>*Intervention process.* Participants will enrol and take part in the intervention, including a first meeting where they learn how to use the online platform, followed by online self-studies at home on their digital devices for nine weeks. Participants are encouraged to complete one course module per week, and each module ends with a brief online evaluation. Module completion is monitored by the course leader/interventionist so that participants stay on pace with the course completion date. The course leader/interventionist is available for questions about homework and practical exercises and provides written encouragement to facilitate course completion.<br>Participants are taught and learn about …<br>Week 1: … light, practical exercises (e.g., observer-based assessment of a room).<br>Week 2: … electric lighting, practical exercises (e.g., a lighting inventory using a checklist).<br>Week 3: … daylight, practical exercises (e.g., measuring daylight levels using a light meter app on the phone).<br>Week 4: … the benefits of daylight exposure and physical activity outdoors. They write an activity diary for one week.<br>Week 5: … planning and implementing a walking programme. Preparatory work for the subsequent sleep modules: they keep a one-week sleep diary.<br>Week 6–9: … environmental cues for setting the body clock, the sleep drive, age-related changes in sleep, the importance of sleep routines, and sleep restriction techniques.<br>In the third and fifth weeks, the participants attend two physical meetings at the senior citizen meeting point in the municipality (one group in municipality A, another group in the adjacent municipality B). A final meeting takes place at the same physical location after the participants have completed the intervention.
5. Who provides?	A researcher (PI) with teaching experience and expertise in lighting and environmental psychology provides feedback to the participants on the digital platform and facilitates the physical meetings at the senior citizen meeting point. A research assistant gives digital support at the first meeting and take

	notes at the following meetings. The municipality provides the locality for the physical meetings (one of the senior citizen meeting points).
6. How?	The intervention delivery is web-based in the form of an online course that participants take individually at home. In addition, physical face-to-face meetings in a group take place on four occasions at the senior citizen meeting point.<br>The course leader/interventionist delivers written encouragement as texts on the phone and written feedback on the digital platform.
7. Where?	In the home on a digital device (computer or tablet) with an internet connection and at a senior citizen meeting point in the municipality.
8. When and how much?	Nine weekly modules are successively unlocked during nine weeks (light modules during weeks 1–3, physical activity modules during weeks 4 and 5, sleep modules during weeks 6–9). Participants can choose when to do the coursework during each weekly module but must follow the course materials sequentially. The estimated course time per week is between 2 and 4 hours, including reading, listening and doing the practical exercises. Estimated time for completing one module is 2 –3 hours. A course module is unlocked after completion of the preceding module to ensure participants complete all modules.
9. Tailoring	The intervention is participant-centred. It addresses self-identified needs, which can make the intervention more effective. Participants decide and act on their own goals concerning changes in the home environment and daily activity and sleep patterns, for example, the type of physical outdoor activity, amount and frequency, or the extent of self-adjustments in their home.
10. Modifications	Not applicable as this checklist is developed for a study protocol.
11. How well? (adherence / fidelity)	Not applicable.

Reference

Hoffmann, T. C., Glasziou, P. P., Boutron, I., Milne, R., Perera, R., Moher, D., Altman, D. G., Barbour, V., Macdonald, H., Johnston, M., Lamb, S. E., Dixon-Woods, M., McCulloch, P., Wyatt, J. C., Chan, A. W., & Michie, S. (2014). Better reporting of interventions: template for intervention description and replication (TIDieR) checklist and guide. *BMJ (Clinical research ed.), 348*, g1687. https://doi.org/10.1136/bmj.g1687
