# Supplementary material for: A municipality implemented behavioural intervention to improve quality of life among older adults: protocol for a mixed-methods pilot case study
Source: Pilot Feasibility Stud. 2026 Mar 14;12:47. doi: 10.1186/s40814-026-01795-w (PMC13063510; doi:10.1186/s40814-026-01795-w)
Supplement: Supplementary file 3 — Additional file 3. Intervention breakdown and evaluation timeline. [file 40814_2026_1795_MOESM3_ESM.pdf]

Additional file 3: Intervention breakdown and evaluation timeline

Recruitment of intervention participants

Municipal staff working at the senior citizen meeting points will be informed about the study so they can assist with participant recruitment. Intervention participants will also be recruited through advertising in the local newspapers and pensioner associations. Participants will be selected who meet the inclusion criteria. Selected participants will be phoned to confirm background information (e.g., Swedish speaking skills). Invitations to meetings will be sent out and meetings will take place at the senior citizen meeting point. (see **Figure 1**).

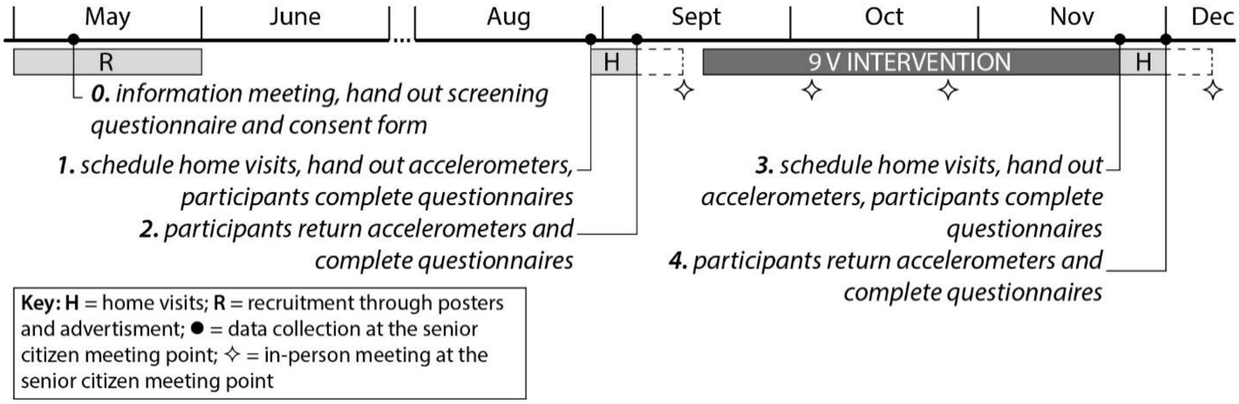

Figure 1. Recruitment and intervention delivery (municipalities A and B, 2024; municipalities C and D, 2025)

Intervention delivery

Participants will enrol and take part in the intervention, including a first introductory meeting when they will learn how to use the online platform (which does not collect sensitive personal data), online self-studies, and three additional in-person meetings at the senior citizen meeting point. The participants submit three assignments on the digital platform but not the 'home assignments' as the purpose is for the participants to self-reflect. **Table 1** shows a breakdown of the intervention into weekly content and activities at the in-person meetings at the senior citizen meeting point.

Table 1. The 'Light, activity and sleep in my daily life' intervention.

**Course week** #	**Individual activities and self-studies at home** (including read/watch/listen and practical exercises)	**Group activities at the physical meetings***	**Course leader/ interventionist** (tasks)
	Course enrolment (a student account at the university is activated by the participant which gives them access to the online course on the digital platform).	–	Sends enrolment instructions to participants by e-mail and reminds them to bring their digital devices (either a computer or tablet) to the first meeting.

<table>
<tr><td>0</td><td>Introduction, including descriptions of the target group, background, course goals and content, assignments, navigation on the digital platform, personal data is processed in accordance with the EU's General Data Protection Regulation (GDPR).
Assignment on the digital platform: ‘Your course expectations’</td><td>Course introduction week:
Physical meeting #1 (90 min).
• Participants are informed that they can access the online content for two additional months after the course has ended.
• Participants are guided and learn (using their own devices) how to navigate, use various functions on the digital platform and communicate with the course leader/interventionist during the course.</td><td rowspan="7">• Encourages participants who want to change bulbs in ceiling-mounted luminaires or hang new curtains to use the minor home help service (in Swedish: Fixartjänst) provided by the municipality free of charge.
• Tracks the participants’ logged-in time and completion of modules. Participants who have not shown any online activity or completed the module receive supporting text messages on the phone to encourage them to stay on pace (one module per week). Participants who have completed the module receive messages congratulating them on their progress.
• Provides written feedback to participants’ comments in the weekly evaluation forms Mondays.
• Provides written feedback to participants’ assignments on the digital platform.
• Facilitates the in-person discussions at meetings.</td></tr>
<tr><td>1</td><td>Module on Light: the effect of light on body and mind.
Assignment on the digital platform: ‘Inventory of a room at home’.</td><td rowspan="2"></td></tr>
<tr><td>2</td><td>Modul on Electric lighting: participants learn about what factors influence the lighting in a room and how it is perceived.
Home assignments: ‘Inventory of the lighting with checklist’; ‘What changes would you like to make?’
Practical exercises: ‘The cap test’; ‘The sheet test’; ‘Changing light sources’.</td></tr>
<tr><td>3</td><td>Modul on Daylight: participants learn about how daylight in a room can be modified to some extent by the occupants.
Practical exercise: ‘Daylight measurement with the mobile phone’.
Home assignment: ‘What changes would you like to make?’</td><td rowspan="3">3rd course week:
Physical meeting #2 (2 hours).
• Participants discuss in groups of two any identified light-related problems in their home environment. They then present their small-group discussions to the large group.
• Participants discuss in groups of two any potential or implemented design solutions to their light-related problems in their home environment. They then present their small-group discussions to the whole group.</td></tr>
<tr><td>4</td><td>Modul on Activity outdoors: participants learn about the benefits of exposure to daylight outdoors and the health benefits of combining outdoor time with walking as a physical activity.
Home assignments: ‘Activity diary for activities outside the home’; ‘Reflection questions’.</td></tr>
<tr><td>5</td><td>Module on Activity and restoration: participants learn about different types of outdoor walking and put together a walking program.
Home assignments: ‘Put together your own program of regular outdoor activity’; ‘Write a sleep and behavior diary’.</td></tr>
<tr><td>6</td><td>Module on Sleep from different perspectives: participants learn about biological and psychological aspects of sleep, and the effect of a physical environment conducive to good sleep.</td><td>6th course week:
Physical meeting #3 (2 hours).
• Participants discuss in groups of two a selection of technical characteristics of distributed lamp packages. They then present the</td></tr>
</table>

	Home assignments: Analysis of last week's sleep diary; Test your sleep deficit; 'Measures to improve sleep'.	characteristics to the whole group.<br>• Participants discuss in groups of two any changes they would like to make regarding physical activity and sleep. They then share with the whole group.	
7	Module on *Sleep later in life:* participants learn about age-related changes to sleep.<br>Home assignment: 'Continued measures to improve sleep'.		
8	Module on *Sleep and routines*: participants learn about the benefit of maintaining sleep-related routines.<br>Assignment on the digital platform: 'Test if you are a morning or evening person'; 'Continued measures to improve sleep'.		
9	Module on *Sleep:* participants learn about the benefit of creating 'if-then plans' for sustaining changes.<br>Home assignments: 'Continued measures to improve sleep'; 'Simple quiz about your sleep'; 'Write a plan in your course journal to maintain changed routines'.		
10	The course ends but the participants can access the online content for two additional months.	Final course week:<br>Physical meeting #4 (90 min).<br>• Participants give their final course evaluation by writing their responses to two questions ('a minute paper' and 'muddiest point'): 1) The most important thing they learnt in the course, 2) Something important that remains answered or you still do not understand.	

* 2024: 10 participants met at the senior citizen point in municipality A on the first day. The following day, 10 other participants met at the senior citizen point in municipality B. During 2025, the procedure will be repeated in municipalities C and D.

Data collection

The usability of the online intervention content and intervention usefulness will be evaluated by intervention participants after the intervention. Participants will give their feedback in audio-recorded face-to-face interviews with open-ended questions at home and self-administered paper-and-pencil questionnaires with closed-ended questions at the senior citizen meeting point (see **Figure 1**).

Intervention outcome measures within participants before and after the intervention will be compared, e.g., quality of life and self-managed changes in the home. Participants will meet at the senior citizen meeting point to fill out questionnaires after giving their informed written

consent and learn to use the accelerometers that will be worn on the wrist for seven subsequent days. Accelerometry will be collected from 10 participants in one municipality during the first week and 10 in another municipality the following week. Home visits will be scheduled during the same period for a face-to-face interview and a home walk-through to observe environmental features (e.g., window openings and their treatment, home lighting, surface colours, and furniture placement). Questionnaire data, interview data (e.g., asking about the participant's daily activities), and accelerometry will be collected before the intervention starts and immediately after delivery. Questionnaire, interview, and accelerometry will be collected again after three and ten months (see **Figure 2**).

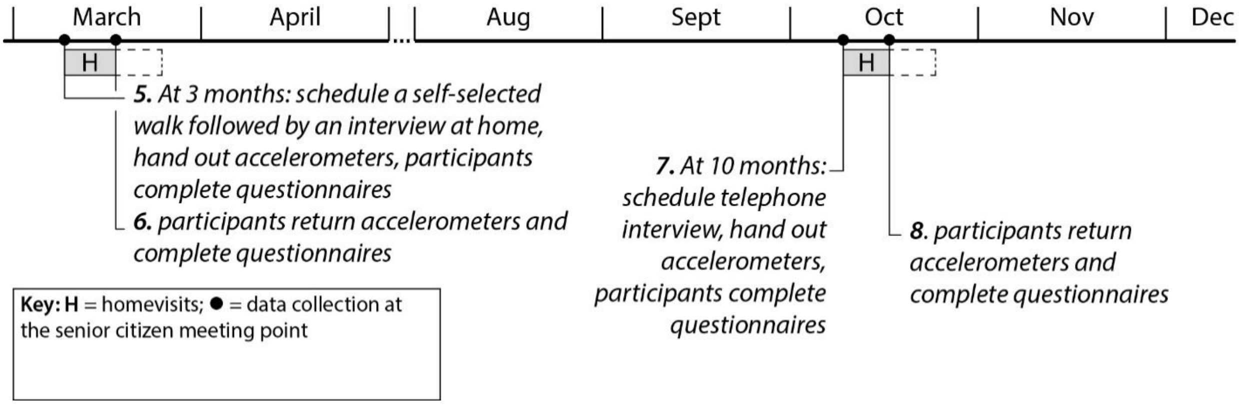

Figure 2. Repeated intervention outcome measures at three and ten months (municipalities A and B, 2025; municipalities C and D, 2026)

Intervention delivery procedures will be evaluated for their acceptance by municipal staff involved in the project (potential service providers). Possibilities for future implementation will also be discussed. They will give their feedback in focus group discussions (6 to 8 participants in each municipality, e.g., staff who have helped recruit intervention participants or assisted intervention participants at the senior citizen meeting points) (see **Figure 3**). The focus group, with a duration of 90 minutes, will be led by one moderator facilitating the discussion and one assistant observing group interactions, recording the discussion and organising coffee breaks. The focus group discussions will be conducted after the intervention.

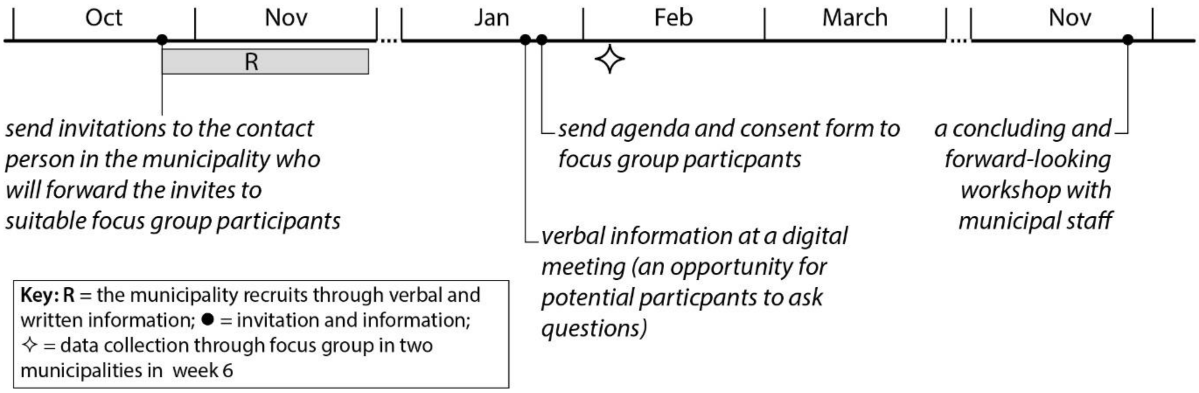

Figure 3. Timeline: focus group (municipalities A and B, 2024–2025; municipalities C and D, 2025–2026)

Enablers and inhibitors to daytime outdoor walking will be identified by intervention participants using semi-structured video-elicitation interviews with open-ended questions three months after the intervention. Video-elicitation interviews will use the following protocol. Course participants, who have completed the intervention, will be shadowed as they take one self-selected walk. The researcher will record environmental features along the walk route, and the participants will video record the walk using body-worn cameras. Video recordings will be shown on a tablet in the participant's home after the walk to assist recollection while the participant thinks aloud about the walk. The purpose is to elicit more information during the interview.

Communication and dissemination

The knowledge produced in the project will be communicated to several research communities (sleep, light and health, public health), practitioners and a general audience. Popular presentations about the results will be given at senior citizen meeting points, targeting a mixed audience of older people and health practitioners employed by the municipalities and regions. The researchers will organise a workshop with staff from the municipalities to discuss whether the intervention has implementation potential and how the intervention might change practice. The discussions will be supported by the *Checklist for high-quality implementation* produced by the Public Health Agency (2024).

Reference

Public Health Agency of Sweden. (2024). *Checklist for high-quality implementation: From news to everyday use – the difficult art of implementation, version 3.0.* https://www.folkhalsomyndigheten.se/publikationer-och-material/publikationsarkiv/f/from-news-to-everyday-use-the-difficult-art-of-implementation/
