# Supplementary material for: A municipality implemented behavioural intervention to improve quality of life among older adults: protocol for a mixed-methods pilot case study
Source: Pilot Feasibility Stud. 2026 Mar 14;12:47. doi: 10.1186/s40814-026-01795-w (PMC13063510; doi:10.1186/s40814-026-01795-w)
Supplement: Supplementary file 4 — Additional file 4. Usability testing questionnaire (SUS). [file 40814_2026_1795_MOESM4_ESM.pdf]

Additional file 4: Usability testing questionnaire (System Usability Scale)

For each of the following statements, please mark one box that best describes your reactions to the online course.

1. I think that I would like to use the online course frequently.

Strongly disagree ☐ ☐ ☐ ☐ ☐ Strongly agree

2. I found the online course unnecessarily complex.

Strongly disagree ☐ ☐ ☐ ☐ ☐ Strongly agree

3. I thought the online course was easy to use.

Strongly disagree ☐ ☐ ☐ ☐ ☐ Strongly agree

4. I think that I would need the support of a technical person to be able to use the online course.

Strongly disagree ☐ ☐ ☐ ☐ ☐ Strongly agree

5. I found the various functions in the online course were well integrated.

Strongly disagree ☐ ☐ ☐ ☐ ☐ Strongly agree

6. I thought there was too much inconsistency in the online course.

Strongly disagree ☐ ☐ ☐ ☐ ☐ Strongly agree

7. I would imagine that most people would learn to use the online course very quickly.

Strongly disagree ☐ ☐ ☐ ☐ ☐ Strongly agree

8. I found the online course very cumbersome (awkward) to use.

Strongly disagree ☐ ☐ ☐ ☐ ☐ Strongly agree

9. I felt very confident using the online course.

Strongly disagree ☐ ☐ ☐ ☐ ☐ Strongly agree

10. I needed to learn a lot of things before I could get going with the online course.

Strongly disagree ☐ ☐ ☐ ☐ ☐ Strongly agree

11. Overall, I would rate the user friendliness of this product as:

☐ Worst imaginable
☐ Awful
☐ Poor
☐ Fair
☐ Good
☐ Excellent
☐ Best imaginable

Comments (optional):
