# Supplementary material for: A municipality implemented behavioural intervention to improve quality of life among older adults: protocol for a mixed-methods pilot case study
Source: Pilot Feasibility Stud. 2026 Mar 14;12:47. doi: 10.1186/s40814-026-01795-w (PMC13063510; doi:10.1186/s40814-026-01795-w)
Supplement: Supplementary file 5 — Additional file 5. Semi-structured interview guide (no. 1–2). [file 40814_2026_1795_MOESM5_ESM.pdf]

Additional file 5: Semi-structured interview guide (no. 1–2)

The following questions (and follow-up questions based on the responses) on the first home visit are used for: mapping the current situation in terms of the participant's home environment and everyday routines, as well as supplementing the survey question about the participant's perceived mood in recent days. Also, information about daily routines can facilitate the analysis of activity and rest patterns through accelerometry, for example if registered daytime rest can be due to naps, meditation or cold baths. On the second home visit after the intervention the questions are used for obtaining participants' in-depth perceptions of the intervention, their thoughts on the usability aspects of the web-based course accessed on their own devices at home, and for reports of any changes to routines or in the home. Questions #2–12 are adapted from Nie *et al.* (2020). The interviews are audio recorded to enable transcription and further analysis.

First interview at home (before the intervention), estimated duration 30–40 minutes	Second interview at home (immediately following the intervention), estimated duration 30 minutes
Background questions<br>A. How long have you lived here in the apartment?<br>B. How many people live in the household?<br>C. Where were you born? How long have you lived in Sweden?<br>D. What is your highest level of education? (Elementary school, residential college for adult education or similar/secondary school /vocational school or similar/university or college)<br>E. What is your current main occupation?<br>F. (If retired) What was your main occupation before retirement?<br>G. Have you worked with:<br>a) course development or pedagogy<br>b) health in general<br>c) specific light, physical activity or sleep	First, I have a few questions about your mood (as a supplement to the mood survey).<br>1. How have you been feeling these past few days - good-bad/drowsy-energetic? Sometimes or very often? [*If 'bad and tired'*] How come, do you think?<br>*Your devices*<br>2. What digital equipment did you use (PC, Mac, tablet etc)?<br>3. Which Internet browser did you use?<br>*General opinions*<br>4. Please, give me your general impression of the online course "Light, activity and sleep".<br>5. What did you like the most about the online course?<br>6. What did you like the least about the online course?
Your routines and the home environment<br>1. Can you describe what a typical day this week at home looks like from the time you wake up to the time you go to bed? [*Fill in the activity bar on the next page*]<br>We can start with the morning… where are you… alarm clock… what activities? Morning/midday/afternoon/evening... what activities?<br>2. Where do you spend the most time in the home when you are awake? Do you have a favorite place?<br>3. I see that there are curtains/blinds/blinds here. When do you use them?<br>4. What routines do you have before you go to sleep? … blackout … opened window … use of any digital screen before going to sleep …<br>5. How did you think when you chose the colours or materials for furniture, e.g. the dining table?<br>6. How much time did you spend outdoors on a typical day during the past week? Approximately what time of day are you outdoors?	*Perceived ease of use*<br>7. Did you find the online course's digital platform easy to use?<br>8. Did you come across any difficulties or challenges?<br>9. Was the graphic design and layout clear?<br>10. Were the "practical exercises"/instructions difficult to follow?<br>11. How do you feel about the overall effort to get through the online course?<br>*Perceived usefulness*<br>12. Has the course been useful for you? How?<br>13. Has the course helped you carry out minor adaptations in your home related to lighting? How? [*Possible follow-up questions*] Any other changes in the room since the last interview? For example, repainted, acquired more or replaced some furniture or some textiles? How did you think about your choices in that case?<br>14. Has the course helped you make minor adaptations in your home related to darkness? How?<br>15. Has the course helped you change your routines related to outdoor activity? How? [*Possible follow-up questions*] How much time did you spend outdoors on a

Physical activity: Your routines and the home environment

7. What is your current level of physical activity?
   a. What do you do during a day or a week?
   b. Is any strength or cardio training included?
8. What type of physical activity do you enjoy because it is fun or otherwise satisfying?
   a. How come?
   b. Do you have previous positive experiences with physical activity?
9. What type of physical activity do you dislike because it is boring or otherwise unsatisfying?
   a. How come?
   b. Do you have previous negative experiences with physical activity?
10. How much of your time are you sedentary? [*If necessary, the person can describe a typical day*]

Mood

11. How have you been feeling these past few days - good-bad/drowsy-energetic? Sometimes or very often? [*If 'bad and tired*'] How come, do you think?

Readiness to change (closing question)

12. Would you say you are …
    … not ready to make any changes in the next 6 months,
    … thinking about making any changes in the next 6 months,
    … thinking about making any changes in the next month?
    Or have you already made any changes (action)?

The interview is completed, and I would like to finish by recording some observations on a form regarding materials, colors and dimensions of the window opening. Is that okay?

Thanks for your participation!

typical day during the last week? Approximately what time of day are you outdoors?

16. Has the course helped you change your routines related to sleep? How?
17. Do you think the course could be useful to others?

Concerns

18. Are there additional features of the online course that you would like to have?
19. Are there features of the online course that you find unnecessary?
20. Have you thought of anything else that I haven't covered?

Closing question

21. How satisfied are you with what you learned?

Thanks for your participation!

[The activity bar including numbers representing the time of day is completed by the researcher during the interview to visualise activity and rest patterns during a normal day over the past week]

1	2	3	4	5	6	7	8	9	10	11	12

13	14	15	16	17	18	19	20	21	22	23	24

Reference

Nie, Q., Nguyen, L. T., Myers, D., Gibson, A., Kerssens, C., Mudar, R. A., & Rogers, W. A. (2020). Design guidance for video chat system to support social engagement for older adults with and without mild cognitive impairment. *Gerontechnology, 20*(1), 1–15. https://doi.org/10.4017/gt.2020.20.1.398.08
