# Supplementary material for: A municipality implemented behavioural intervention to improve quality of life among older adults: protocol for a mixed-methods pilot case study
Source: Pilot Feasibility Stud. 2026 Mar 14;12:47. doi: 10.1186/s40814-026-01795-w (PMC13063510; doi:10.1186/s40814-026-01795-w)
Supplement: Supplementary file 6 — Additional file 6. Focus group interview guide. [file 40814_2026_1795_MOESM6_ESM.pdf]

Additional file 6: Focus group interview guide

After intervention delivery, staff from the municipality health care service (6–8 per municipality) will participate in focus groups to discuss the acceptance of intervention delivery and future implementation. For example, the discussion includes any identified challenges and thoughts on how the intervention could be integrated into municipal services in the future. The discussions are audio-recorded to enable transcription and further analysis. The focus group will be led by one moderator, who will facilitate the discussion, and one assistant, who will observe group interactions, record the discussion, and organise coffee breaks.

Focus group, estimated duration 90 minutes

"Could we start with a short presentation round, where you state your first name, whether you have interacted with the course participants, and at what stage (during recruitment and/or when the course was delivered)?"

1. "Let's move on to the discussion topics."

Recruitment:

a) How do you think the recruitment went?

Reactions to the web-based course implementation (according to your observations and experiences):

b) How do you perceive the participants have reacted to the Internet course – if you have come into contact with them?

c) Have any aspects been particularly successful?

d) Have any been particularly challenging?

Possibility of future implementation and identified barriers:

e) What do you think about the possibility of delivering the course as a service in the municipality in the future?

f) What do you see as opportunities and challenges with such an initiative?

g) Are there, for example, specific resource shortages or organisational obstacles that may affect the implementation?

h) Is there a need for additional training or resources for employees? Are there opportunities for collaboration with other departments or external resources to support implementation?

2. Summarise and ask the participants if there is anything we have overlooked.

[*Turn off the audio recording*]

Closing. Inform them about the next step in the project – that we will meet again at the workshop in November, where we will also present the project's preliminary results. Thank them for participating.

Thanks for your participation!

Reference

Public Health Agency of Sweden/Folkhälsomyndigheten. (2023). Checklist for high-quality implementation. From news to everyday use – the difficult art of implementation. https://www.folkhalsomyndigheten.se/contentassets/b4134ae1187a4578ba9712e67c2b7cc5/checklista-implementering-kvalitet.pdf
