# Supplementary material for: A municipality implemented behavioural intervention to improve quality of life among older adults: protocol for a mixed-methods pilot case study
Source: Pilot Feasibility Stud. 2026 Mar 14;12:47. doi: 10.1186/s40814-026-01795-w (PMC13063510; doi:10.1186/s40814-026-01795-w)
Supplement: Supplementary file 7 — Additional file 7. Semi-structured interview guide (no. 3–4). [file 40814_2026_1795_MOESM7_ESM.pdf]

Additional file 7: Semi-structured interview guide (no. 3–4)

The interview questions (and follow-up questions based on the responses) 3 months after the intervention will supplement the survey question about the participants' perceived mood in recent days, help identify enablers and inhibitors to daytime outdoor walking using video-elicitation and provide reports on whether routines have continued or discontinued since the second interview. Also, information about daily routines can facilitate the analysis of activity and rest patterns through accelerometry, for example, if registered daytime rest can be due to naps, meditation or cold baths. Video elicitation enables an interview at home rather than during the pre-interview outdoor walk. The purpose of the video is to stimulate recall (Lyle, 2003). The video-elicited interviews will use the following protocol. A researcher and an assistant will shadow participants who have completed the intervention as they take one self-selected walk. The researcher will record environmental features along the walk route, and the participants will video-record the walk using body-worn cameras. The video will be shown on a computer screen in the participant's home after the walk to assist recollection while the participant thinks aloud about the walk.

The fourth interview, conducted by phone, will supplement the survey question about the participant's perceived mood in recent days and provide reports on whether routines have continued or discontinued since the third interview.
The interviews are audio-recorded to enable transcription and further analysis.

Third interview at home (3 months after the intervention), estimated duration 60 minutes	Fourth interview on the phone (10 months after intervention), estimated duration 30 minutes
Background questions<br>First, I have a few questions about your mood (as a supplement to the mood survey).<br>1. How have you been feeling these past few days - good-bad/drowsy-energetic? Sometimes or very often? [*If 'bad and tired'*] How come, do you think?<br>Let's move on to your experience of the outdoor environment along the walking route. We will play the video and want you to tell us about your experience of the route simultaneously.<br>[*Start the video*]<br>*The walk*<br>2. Take us through the walk. Please tell us what you like about it and what you don't like.<br>[Pay attention to whether any moment or situation during the walk stands out, and if so, ask the participant to comment.]<br>[*End the video*]<br>*Perceived enablers and barriers*<br>3. Why did you chose this particular route?<br>4. Do you have several walks to choose from?<br>5. Would you have chosen a different route if you had a different goal for your walk (e.g., exercise, errand)?<br>*Continued or discontinued walking*<br>6. Is walking something you would like to continue doing?<br>7. What would make you walk more? What makes you refrain from taking a walk?<br>*Physical activity in general*<br>8. Do you do physical activity other than walking? (e.g. outdoor gym, water gymnastics, pilates)	First, I have a few questions about your mood (as a supplement to the mood survey).<br>1. How have you been feeling these past few days - good-bad/drowsy-energetic? Sometimes or very often? [*If 'bad and tired'*] How come, do you think?<br><br>I would like to ask about your routines and whether they have changed since we last met.<br>2. Have you changed your routines connected to outdoor physical activity since the last interview? In what way? How much time do you spend outdoors? Roughly at what time are you outdoors? How come?<br>3. Have you changed your sleep routines? In what way?<br>4. Is there anything else about your routines related to light, activity and sleep that you would like to address?<br><br>Thanks for your participation!

9. Have you changed your routines connected to outdoor physical activity since the last interview? In what way?

Outdoor time in general

10. Do you stay outdoors at times other than when you walk? (E.g., sitting outside in the courtyard or on your allotment.)
11. How much time do you spend outdoors?
12. Roughly at what time are you outdoors? How come?

I would also like to ask a few questions about your *sleep routines*.

13. Have you changed your sleep routines since we last met? In what way?
14. Is there anything else about your routines related to light, activity and sleep that you would like to address?

Thanks for your participation!

Reference

Lyle, J. (2003). Stimulated Recall: a report on its use in naturalistic research. *British Educational Research Journal, 29*(6), 861–878.
