# Supplementary material for: A municipality implemented behavioural intervention to improve quality of life among older adults: protocol for a mixed-methods pilot case study
Source: Pilot Feasibility Stud. 2026 Mar 14;12:47. doi: 10.1186/s40814-026-01795-w (PMC13063510; doi:10.1186/s40814-026-01795-w)
Supplement: Supplementary file 8 — Additional file 8. Environmental outdoor features form. [file 40814_2026_1795_MOESM8_ESM.pdf]

Additional file 8: Environmental outdoor features form

The observation form is used to document and observe the physical and social environment along a walk route. The researcher records characteristic features in the form while shadowing a participant during a self-selected daytime walk videotaped by the participant wearing a body-worn camera. The observations will serve as a supplemental interview guide during the subsequent video-elicited interview in the participant's home (see Additional file 5). The final report will use selected observations to describe essential characteristics of the urban outdoor areas where participants take their daily walks.

Municipality:	Date:
Participant ID:	Observer:
Weather and temperature:	Start time:
Residential or mixed-use district:	End time:
Peripheral, mid, central:	Duration of walk:

**Timestamp**	**Observations, including comments**

**Stationary features**	**Moving features**
Bench **(BE)**	People **(PE)**
Pavement/Footpath/Pedestrian and cycle paths **(PA) (FP) (PC)**	Animal **(AN)**
Separation strip between pedestrian and cycle lane **(SS)**	Skateboard, inlines **(SK)**
Pedestrian kerb ramp by crossing **(PKR)**	Cyclists **(CY)**
Walking surface (accessibility, unevenness, bare ground, slush snow, snow) **(WS)**	Electric scooter **(EL)**
Safety barriers for pedestrians (sidewalk buffers, green strip of vegetation, posts, parking spaces) **(SB)**	Cargo bike **(CA)**
Crossing (signed, painted, signaled) **(CR)**	Motor vehicle **(MO)**
Junction/intersection (traffic signals, road signs) **(JU)**	Tram **(TR)**
Greenery along sidewalk/walking paths (trees, vegetation, abundant, sparse) **(GR)**	Railway **(RA)**
Green spaces (parks, greenery in the neighborhood) **(GS)**	Sound **(SO)**
Service facilities (grocery store, café, hairdresser, health care centre, senior meeting point, training fitness facility/wellness centre) **(SF)**	
Transit stops for public transport (transit shelter with/without roof/side walls) **(TS)**	
Graffiti and damage (clear deficiencies in repair and maintenance) **(GR)**	
