# Supplementary material for: A municipality implemented behavioural intervention to improve quality of life among older adults: protocol for a mixed-methods pilot case study
Source: Pilot Feasibility Stud. 2026 Mar 14;12:47. doi: 10.1186/s40814-026-01795-w (PMC13063510; doi:10.1186/s40814-026-01795-w)
Supplement: Supplementary file 9 — Additional file 9. Intervention outcome measures. [file 40814_2026_1795_MOESM9_ESM.pdf]

Additional file 9: Intervention outcome measures

Material

Table 1. Intervention outcome measures.

Category	Method of evaluation	Reference
Quality of life[1]	Self-reported: WHOQOL-BREF questionnaire	(1)
Mood[1]	Self-reported: Short Swedish Core Affect Scales (sSCAS)	(2)
Sleep quality[1]	Self-reported: PROMIS 4-item Sleep Disturbance instrument	PROMIS Item Bank v1.0 (3)
Behavioural skill[1]	Self-reported: A New General Self-efficacy Scale	(4,5)
Computer anxiety[2]	Self-reported: Short Computer Anxiety Scale	(6)
Lighting quality[2]	Self-reported: Perceived indoor lighting quality (PILQ)	Derived from outdoor lighting scales (7)
Sleep (activity and rest patterns) [1]	Performance-based measure using a wrist-worn accelerometer (ActiGraph wGT3X-BT, ActiGraph LLC, Pensacola, FL).	
Sleep times[1]	Self-reported: bedtime (turning lights out to go to sleep) and final awakening time using a brief sleep diary to be used as sleep period start and end times in the sleep analysis.	
Physical activity, sedentary behaviour and steps[1]	Performance-based measure using a wrist-worn accelerometer with an in-built photopic light sensor (ActiGraph wGT3X-BT, ActiGraph LLC, Pensacola, FL).	

[1] At four timepoints: baseline, after the intervention, at 3 and 10 months.
[2] At two timepoints: before and after the intervention.

Table 2. Questions and items in questionnaires measuring intervention outcomes.

Category	Question or statement	Response format
Quality of Life	How would you rate your quality of life?	1 = Very poor; 2 = Poor; 3 = Neither poor nor good; 4 = Good; 5 = Very good
	How satisfied are you with your health?	5 = Very dissatisfied; 4 = Dissatisfied; 3 = Neither satisfied nor dissatisfied; 2 = Satisfied; 1 = Very satisfied
	In the last two weeks:	
	To what extent do you feel that (physical) pain prevents you from doing what you need to do?	1 = Not at all; 2 = A little; 3 = A moderate amount; 4 = Very much; 1 = An extreme amount
	How much do you need any medical treatment to function in your daily life?	
	How much do you enjoy life?	
	To what extent do you feel your life to be meaningful?	
	How well are you able to concentrate?	1 = Not at all; 2 = A little; 3 = A

	How safe do you feel in your daily life?<br>How healthy is your physical environment?	moderate amount; 4 = Very much;<br>5 = Extremely
	Do you have enough energy for everyday life?<br>Are you able to accept your bodily appearance?<br>Have you enough money to meet your needs?<br>How available to you is the information that you need in your day-to-day life?<br>To what extent do you have the opportunity for leisure activities?	1 = Not at all; 2 = A little; 3 = Moderately; 4 = Mostly;<br>5 = Completely
	How well are you able to get around?	1 = Very poor; 2 = Poor; 3 = Neither poor nor good; 4 = Good; 5 = Very good
	How satisfied are you with your sleep?<br>How satisfied are you with your ability to perform your daily living activities?<br>How satisfied are you with your capacity for work?<br>How satisfied are you with yourself?<br>How satisfied are you with your personal relationships?<br>How satisfied are you with your sex life?<br>How satisfied are you with the support you get from your friends?<br>How satisfied are you with the conditions of your living place?<br>How satisfied are you with your access to health services?<br>How satisfied are you with your transport?	1 = Very dissatisfied; 2 = Dissatisfied;<br>3 = Neither satisfied nor dissatisfied;<br>4 = Satisfied; 5 = Very satisfied
	How often do you have negative feelings such as blue mood, despair, anxiety, depression?	1 = Never; 2 = Seldom; 3 = Quite often; 4 = Very often; 5 = Always
Mood	How have you mostly felt for the past days?<br>(two scales including three adjective pairs measuring the dimension 'valence', i.e. good or bad, and three measuring 'activation')	1- to 9-point scale:<br>'Very sad/dull/depressed' to 'Very glad/peppy/joyful';<br>'Very passive/displeased/sleepy' to 'Very active/pleased/awake'
Sleep quality	In the past seven days, my sleep quality was …	5 = Very poor; 4 = Poor; 3 = Fair; 2 = Good; 1 = Very good
	In the past seven days …:<br>… my sleep was refreshing<br>… I had problem with my sleep (reversed scoring)<br>… I had difficulty falling asleep (reversed scoring)	5 = Not at all; 4 = A little bit; 3 = Somewhat; 2 = Quite a bit; 1 = Very much
Behavioural skill	Eight statements on: goal achievement, task accomplishment, obtaining important outcomes, succeeding at most any endeavour, overcoming challenges, effective performance, doing tasks well, performing well even when things are tough	Strongly disagree = 1; Disagree = 2; Neither agree nor disagree =3; Agree = 4; Strongly agree = 5
Computer anxiety	Eight statements, such as 'The harder I work at learning computers the more confused I get'; 'I have sometimes thought "Computers don't like me"; 'I can usually manage to solve computer problems by myself' (reversed scoring)	1 = Strongly disagree; 2 = Disagree; 3 = Mildly disagree; 4 = Mildly agree; 5 = Agree; 6 = Strongly agree
Lighting quality	Five bipolar scales measuring the dimension 'Strength' and five measuring the dimension	1- to 7-point scale:<br>'Dark' to 'Light'; 'Weak' to 'Strong';

'Hedonic tone': How do you perceive the light in this room?	'Unfocused' to 'Focused'; 'Drab' to 'Clear'; 'Subdued' to 'Brilliant'; 'Cool' to 'Warm'; 'Hard' to 'Soft'; 'Unnatural' to 'Natural'; 'Sharp' to 'Mild'; 'Glaring' to 'Shaded'
How well can you see in this light?	1- to 7-point scale: 'Very poorly' to 'Very good';

Quality of Life scores on 26 items are summed in each domain (after three negatively phrased items have been reversed), after which they are converted to raw scores and then transformed scores. Participants' scores from the PROMIS 4-item Sleep Disturbance instrument are summed, resulting in a raw score ranging from 4 to 20. The raw score are then translated to a total raw score (T-score) ranging from 32 to 73.3 using a conversion table. Participants' scores on eight items in the New General Self-Efficacy Scale are summed and divided by eight, resulting in a global score ranging from 1 to 5. Participants' scores on six items in the Short Computer Anxiety Scale are summed, resulting in a global score ranging from 6 to 36. Participants' scores on five items measuring the dimension 'strength' in the Perceived Indoor Lighting Quality (PILQ) questionnaire were summed and divided by five, resulting in a total score, ranging from 1 to 7. The scores on five PILQ items measuring the dimension 'hedonic tone' are summed and divided by five, resulting in a total score, ranging from 1 to 7.

Data analysis of intervention outcome measures

Data from the questionnaires are analysed descriptively in SPSS by comparing individual participants' scores from baseline to after the intervention. Accelerometer-measured activity and rest patterns are analysed using the software ActiLife v613.4 to document changes from baseline by subtracting the post-intervention measurement from the baseline measurement on sedentary behaviour, number of steps and physical activity (light physical activity and moderate-to-vigorous physical activity (MVPA)). The software ActiLife v613.4 are used to analyse changes in sleep behaviour from baseline by subtracting the post-intervention measurement from the baseline measurement (total sleep time (TST), sleep efficiency (SE), wake after sleep onset (WASO) and number of awakenings).

References

1. WHO. Development of the World Health Organization WHOQOL-BREF quality of life assessment. The WHOQOL Group. Psychol Med. 1998;28:551–558.

2. Västfjäll D, Gärling T. Validation of a Swedish short self-report measure of core affect. Scand J Psychol. 2007;48:233–238.

3. PROMIS Sleep Disturbance Scoring manual. https://forms.loinc.org/75258-4 (2023). Accessed 23 Dec 2022.

4. Chen G, Gully SM, Eden D. Validation of a new general self-efficacy scale. Organ Res Methods. 2001;4:62–83.

5. Bandura A. Self-efficacy: The exercise of control. New York: W. H. Freeman; 1997.

6. Lester D, Yang B, James S. A short computer anxiety scale. Percept Mot Skills. 2005;100:964–968.

7. Johansson M, Pedersen E, Maleetipwan-Mattsson P, Kuhn L, Laike T. Perceived outdoor lighting quality (POLQ): A lighting assessment tool. J Environ Psychol. 2014;39:14–21.
